# What about me…? The PVT: a role for the paraventricular thalamus (PVT) in drug-seeking behavior

**DOI:** 10.3389/fnbeh.2013.00018

**Published:** 2013-03-06

**Authors:** Morgan H. James, Christopher V. Dayas

**Affiliations:** ^1^Neurobiology of Addiction Laboratory, School of Biomedical Sciences and Pharmacy, University of NewcastleNewcastle, NSW, Australia; ^2^The Centre for Translational Neuroscience and Mental Heath Research, The Hunter Medical Research InstituteNewcastle, NSW, Australia

The lack of effective pharmacotherapies to prevent relapse to drug taking emphasizes the importance of fully characterizing the brain pathways responsible for this behavior (Kalivas and McFarland, 2003). Recently, there have been attempts to more fully understand the brain circuitry responsible for drug-seeking behavior, beyond the well-characterized nodes such as the prefrontal cortex (PFC), nucleus accumbens (NAC), and ventral tegmental area (VTA). In this respect, the review of Martin-Fardon and Boutrel (2012) is important and timely and should serve to stimulate continued focus on the *paraventricular thalamus* (PVT) in the addiction field. Indeed, their review is an appropriate addition to the recent article “Emerging, re-emerging, and forgotten areas of the reward-circuit” (McGinty et al., 2011).

The first purpose of this commentary is to reiterate this point, but to perhaps go one step further. Thus, in response to the authors' first question (i.e., whether the PVT should be considered part of the drug-seeking circuitry), we argue that there is sufficient anatomical and functional evidence to support this suggestion. For example, the PVT sends glutamatergic projections to the NAC and PFC (Christie et al., 1987; Bubser and Deutch, 1998; Vertes and Hoover, 2008), and a large percentage of these projections are branched, suggesting that a single PVT neuron can influence these areas simultaneously (Otake and Nakamura, 1998). PVT neurons also project to medial, central, and basal nuclei of the amygdala as well as the bed nucleus of stria terminalis (Vertes and Hoover, 2008). Importantly, glutamatergic efferents from the PVT are closely apposed to dopamine fibers in the NAC shell (Pinto et al., 2003) and stimulation of the PVT produces an efflux of dopamine in this brain region (Jones et al., 1989; Parsons et al., 2007). Earlier, lesion and Fos-mapping studies were the first to implicate the PVT as a reward-responsive site. For example, acute psychostimulant administration was found to activate the PVT (Deutch et al., 1998) and lesions of the PVT block the conditioned locomotor response to a cocaine-paired environment (Young and Deutch, 1998). More recent studies have extended these initial findings. Work by McNally's group, and ours, has demonstrated that lesions or chemical inactivation of the PVT suppresses drug-seeking behavior. For example, Hamlin et al. (2009) showed that lesions of the PVT prevent context-induced reinstatement of alcohol-seeking and Marchant et al. (2010) showed that intra-PVT infusion of a κ-opioid receptor agonist also inhibits this behavior. Our group has also shown that inactivation of the PVT using TTX or intra-PVT injections of the inhibitory peptide *cocaine- and amphetamine-regulated transcript* (CART) attenuates cocaine-primed reinstatement (James et al., 2010). This role likely extends to cue-induced cocaine-seeking, as the magnitude of reinstatement behavior is strongly correlated with Fos-activation in the PVT (Dayas et al., 2008; James et al., 2011a). Together, these data strongly support a functional role for the PVT in drug-seeking, however, it will be important for future studies to apply electrophysiological or optogenetic techniques to dissect the circuit-level changes involving PVT efferents onto reward-relevant brain regions (Cao et al., 2011). Designer receptors exclusively activated by designer drugs (DREADD) may also be useful in allowing for selective activation/inactivation of the PVT during reinstatement testing (Dong et al., 2010).

The second question the authors' raise in their review is whether orexin (hypocretin) input within the PVT modulates reinstatement behavior. We agree with the authors that there is strong anatomical evidence implicating the PVT as a site of integration for drug-related hypothalamic signaling. However, the answer to this question appears less straightforward than their more general question regarding the PVT, and we believe that this second issue requires further study—a point acknowledged by Martin-Fardon and Boutrel. The authors cite recent data from their laboratory supporting a role for PVT orexin in reinstatement behavior. PVT infusions of orexin-A reinstated both extinguished cocaine- and sweetened condensed milk-seeking (SCM) behavior. Interestingly, moderate doses of orexin-A produced a stronger reinstatement of cocaine-seeking than for SCM, indicating drug-induced adaptation to orexin receptor expression/function in the PVT (Martin-Fardon et al., 2011). We recently tested the effect of intra-PVT administration of SB-334867, an orexin receptor 1 antagonist, on cue-induced cocaine-seeking. Given our previous demonstration that drug-cue sensitive PVT neurons are closely apposed by orexin terminals (Dayas et al., 2008), it was surprising that microinjections of SB-334867, at a dose also likely to block orexin receptor 2, had no effect on cue-induced reinstatement of cocaine-seeking (James et al., 2011b). In contrast, intra-VTA SB-334867 suppressed drug-seeking behavior (James et al., 2011b), consistent with studies showing that infusions of orexin peptide into the VTA enhance dopamine release in the NAC (Narita et al., 2006; Espana et al., 2010) and reinstate drug-seeking (Wang et al., 2009). Interestingly, we found reduced PVT Fos-expression after intra-VTA SB-334867 infusion, but increased Fos-protein in the NAC shell (James et al., 2012). Previous reports indicate that the NAC shell can exert an inhibitory influence over drug-seeking through its projections to the LH (Millan et al., 2010). Thus, it is possible that intra-VTA SB-334867 reduced PVT recruitment and increased NAC shell inhibitory output to the LH, resulting in attenuated drug-seeking behavior.

How can these apparent contradictory findings relating to orexin signaling in the PVT be reconciled? One plausible explanation is that infusion of orexin-A into PVT may have engendered a stress-like response that evoked drug-seeking. Indeed, Martin-Fardon and Boutrel discuss recent evidence that orexin signaling in this region is important in regulating negative emotional states. For example, Li and colleagues report that intra-PVT infusion of TCSOX229, an orexin receptor-2 antagonist, attenuates the expression of anxiety-like behaviors produced by prior footshock stress (Li et al., 2010) as well as conditioned place aversion produced by precipitated morphine withdrawal (Li et al., 2011). Further, Heydendael and colleagues recently showed that in rats exposed to daily swim stress, orexin-A application increases the responsivity of PVT cells, and intra-PVT infusions of SB-334867 prior to daily swim stress inhibit the ACTH secretion in response to novel stress (Heydendael et al., 2011). Thus, it is possible that the disparate findings regarding orexin signaling in PVT may reflect a preferential role for orexin signaling in stress-induced reinstatement. Future studies using more sophisticated techniques may help resolve this issue.
